# Enhanced melioidosis surveillance in patients attending four tertiary hospitals in Yangon, Myanmar

**DOI:** 10.1017/S095026882100128X

**Published:** 2021-06-22

**Authors:** Mo Mo Win, Kyi Kyi Nyein Win, Thin Thin Wah, Su Nyein Aye, Tin Tin Htwe, Khwar Nyo Zin, Myint Thazin Aung, Wah Wah Aung, Elizabeth A. Ashley, Frank Smithuis, Vanessa Rigas, Bart J. Currie, Mark Mayo, Jessica R. Webb, Clare L. Ling, Zaw Than Htun, David A.B. Dance

**Affiliations:** 1 Department of Medical Research, Yangon, Myanmar; 2 Yangon General Hospital, Yangon, Myanmar; 3 North Okklapa General Hospital, Yangon, Myanmar; 4 Myanmar-Oxford Clinical Research Unit, Yangon, Myanmar; 5 Centre for Tropical Medicine and Global Health, Nuffield Department of Medicine, University of Oxford, Oxford, UK; 6 Menzies School of Health Research, Charles Darwin University, Darwin, Northern Territory, Australia; 7 Shoklo Malaria Research Unit, Mahidol Oxford Tropical Medicine Research Unit, Maesot, Thailand; 8 Lao-Oxford-Mahosot Hospital-Wellcome Trust Research Unit, Vientiane, Lao PDR; 9 Faculty of Infectious and Tropical Diseases, London School of Hygiene and Tropical Medicine, London, UK

**Keywords:** *Burkholderia pseudomallei*, emerging infections, melioidosis, Myanmar

## Abstract

To investigate the current epidemiology of melioidosis in Yangon, Myanmar, between June 2017 and May 2019 we conducted enhanced surveillance for melioidosis in four tertiary hospitals in Yangon, where the disease was first discovered in 1911. Oxidase-positive Gram-negative rods were obtained from the microbiology laboratories and further analysed at the Department of Medical Research. Analysis included culture on Ashdown agar, the three disc sensitivity test (gentamicin, colistin and co-amoxiclav), latex agglutination, API 20 NE, antibiotic susceptibility testing, and a subset underwent molecular confirmation with a *Burkholderia pseudomallei* specific assay. Twenty one of 364 isolates (5.7%) were confirmed as *B. pseudomallei* and were mostly susceptible to the antibiotics used in standard therapy for melioidosis. Ten patients were from Yangon Region, nine were from Ayeyarwaddy region, and one each was from Kayin and Rakhine States. A history of soil contact was given by seven patients, five had diabetes mellitus and one had renal insufficiency. The patients presented with septicaemia (12 cases), pneumonia (three cases), urinary tract infection (two cases) and wound infection (four cases). Eighteen patients survived to hospital discharge. This study highlights the likelihood that melioidosis may be far more common, but underdiagnosed, in more rural parts of Myanmar as in other countries in SE Asia.

## Introduction

*Burkholderia pseudomallei* is a motile, oxidase-positive, Gram-negative, non-fermentative bacillus which causes melioidosis, a glanders-like disease of both humans and animals. It was first described in Myanmar by Whitmore and Krishnaswami in 1911 in a 40-year-old morphine addict who died from acute fulminating pneumonia with superficial abscesses of the leg [1, 2]. The disease is now known to be relatively common in parts of South East Asia and Northern Australia, but a recent modelling study suggested that it may still be far commoner than is currently appreciated, with the model estimating some 165 000 cases and 89 000 deaths worldwide each year [3]. The clinical manifestations of melioidosis are extremely varied, including multisystem involvement with deep visceral abscesses, especially in the liver and spleen, pustular necrotic skin or subcutaneous lesions, pyrexia of unknown origin, septicaemia, central nervous system infections, head and neck infections including parotid abscesses, especially in children and pulmonary infections resembling tuberculosis or pneumonia. Severe melioidosis has a high mortality, which may exceed 70% if patients are not treated appropriately and if ICU facilities are lacking [3, 4].

*Burkholderia pseudomallei* is an environmental saprophyte found in the tropics and in endemic areas is readily isolated from mud and surface water, particularly rice paddy [3]. It can be acquired through inoculation, inhalation or ingestion of soil or water. People engaged in occupations such as farming or gardening in endemic environments have an increased risk of acquiring melioidosis. In endemic areas, approximately 5–30% of healthy adults have evidence of exposure to *B. pseudomallei*, with indirect haemagglutination antibody titres of 1:40 or higher [5, 6]. In addition, people with diabetes mellitus are at greater risk of developing melioidosis, which may lead to the disease becoming more common as the prevalence of diabetes increases in a population [3].

Since the early descriptions of *B. pseudomallei*, when it was said to account for one in every 20 autopsies carried out in Yangon General Hospital [7], melioidosis has been reported rarely in Myanmar. This is probably because it is difficult to diagnose clinically, necessitating microbiological confirmation. Relatively few clinicians and microbiologists are familiar with the disease and its causative organism, which may be discarded as a contaminant, so it frequently goes un- or misdiagnosed [3]. Recently, however, awareness has been increasing and there have been a number of case-reports from the Yangon area over the past 17 years [8–13]. The recent modelling study predicted that more than 6200 cases and 3600 deaths due to melioidosis may occur each year in Myanmar [3], so it is likely that many more cases are still undiagnosed. Many patients may thus be dying unnecessarily because they do not receive appropriate diagnosis and treatment. We therefore undertook a prospective study using simple methods to identify cases of melioidosis in four general hospitals in and around Yangon.

## Methods

### Study sites

We selected four hospitals in Yangon for the study based on their catchment areas (in order to give broad geographical coverage of Yangon) and the presence of in-house microbiology laboratories: Yangon General Hospital (YGH; 2000-bedded hospital located in the centre of Yangon), Thigangyun General Hospital (TGH; 500-bedded hospital located in the eastern part of Yangon), Insein General Hospital (IGH; 500-bedded hospital located in the northern part of Yangon) and North Okklapa General Hospital (NOGH; 800-bedded hospital located in the eastern part of Yangon). The study period was from June 2017 to May 2019.

### Clinical case detection

At the start of the study, physicians from the study sites were provided with information about the clinical features, diagnosis and management of melioidosis through oral presentations in order to raise their awareness of the disease. When isolates were confirmed as *B. pseudomallei*, either by the hospital laboratories or by the Department of Medical Research, Yangon (DMR), the responsible physicians were informed so that patients received the appropriate management for melioidosis. Patients' hospital charts were reviewed for basic socio-demographic and clinical data, including any history of a known exposure event and underlying diseases, which were recorded on a standard proforma (Supplementary material 1).

### Hospital microbiological investigations

Prior to the start of sample collection, meetings were held with the microbiologists from the study hospitals' laboratories to alert them to the study and to highlight the key features of *B. pseudomallei*. Clinical samples were processed according to the normal procedures in the laboratories concerned. Organisms isolated from blood cultures, or in pure or predominant culture from body fluids, pus, sputum or urine, were tested by Gram stain and oxidase test. Isolates suspected of being *B. pseudomallei* (oxidase-positive Gram-negative bacilli that were not obviously *Pseudomonas aeruginosa* based on the presence of green pigment and a typical smell) were stored on nutrient agar slants. These were collected weekly from the microbiology laboratories, along with clinical data proformas, and analysed further at DMR.

### Processing of samples at DMR

The isolates were sub-cultured from the nutrient agar slants onto Ashdown's selective agar and blood agar, incubated at 35–37 °C for 24–96 h aerobically and examined daily. Gram staining and oxidase tests were performed on all isolates. Oxidase-positive Gram-negative rods which grew on Ashdown's agar were screened by disc diffusion for resistance to gentamicin (10 μg) and colistin (10 μg) and susceptibility to co-amoxiclav (20 + 10 μg), and tested by latex agglutination for presumptive identification of *B. pseudomallei* according to the Standard Operating Procedures developed by Wellcome Trust Mahidol University Oxford Tropical Medicine Research Programme [14]. Finally, the identity of isolates that were consistent with *B. pseudomallei* by the screening tests was confirmed by API 20 NE (BioMérieux UK Ltd, Basingstoke, UK). All laboratory procedures at DMR were performed in a Class II Biological Safety Cabinet. The study was assessed against the ‘Microbiology Investigation Criteria for Reporting Objectively (MICRO)’ criteria [15] and complied with the majority of the criteria (Supplementary Table T1).

### Antibiotic susceptibility testing

Isolates that were *B. pseudomallei* latex agglutination positive were tested at DMR for their susceptibility by disc diffusion to ceftazidime (30 μg), meropenem (10 μg), chloramphenicol (30 μg), doxycycline (30 μg), co-amoxiclav and co-trimoxazole (25 μg) using a modification of the Clinical and Laboratory Standards Institute (CLSI) method developed by the Wellcome Trust Mahidol University Oxford Tropical Medicine Research Programme [14] since this study ante-dated the publication of the EUCAST method [16]. Isolates that appeared resistant on initial testing in Yangon were later re-tested using fresh batches of antibiotic discs and were regarded as susceptible if the second tests classified them as such. Seven isolates were also tested against the relevant antibiotics by gradient diffusion (ETEST® BioMérieux) at Menzies School of Health Research, Darwin, Australia, with isolates classified as susceptible or resistant in accordance with the CLSI MIC guidelines 2017 [17].

### Molecular testing

The identities of the first nine latex agglutination positive isolates were confirmed using a *B. pseudomallei* specific real-time PCR assay targeting a 115-bp segment within the Type III Secretion System at the Shoklo Malaria Research Unit, Thailand [18, 19]. The nine clinical isolates and seven soil isolates from Myanmar (two from Magway region, four from Bago region and one from Mon state) were also analysed at Menzies School of Health Research, Darwin, Australia using BOX-PCR [20] and whole genome sequencing (the results of which are reported elsewhere) [21].

### Ethical considerations

Ethical approval was obtained from the Ethics Review Committee of the Department of Medical Research on 16 June 2017 for the study *Detection of Burkholderia pseudomallei in patients attending Yangon, Thingangyun, Insein and North Okkalapa General Hospitals (2017)* (approval number DMR/2017/081).

## Results

**Fig. 1.**
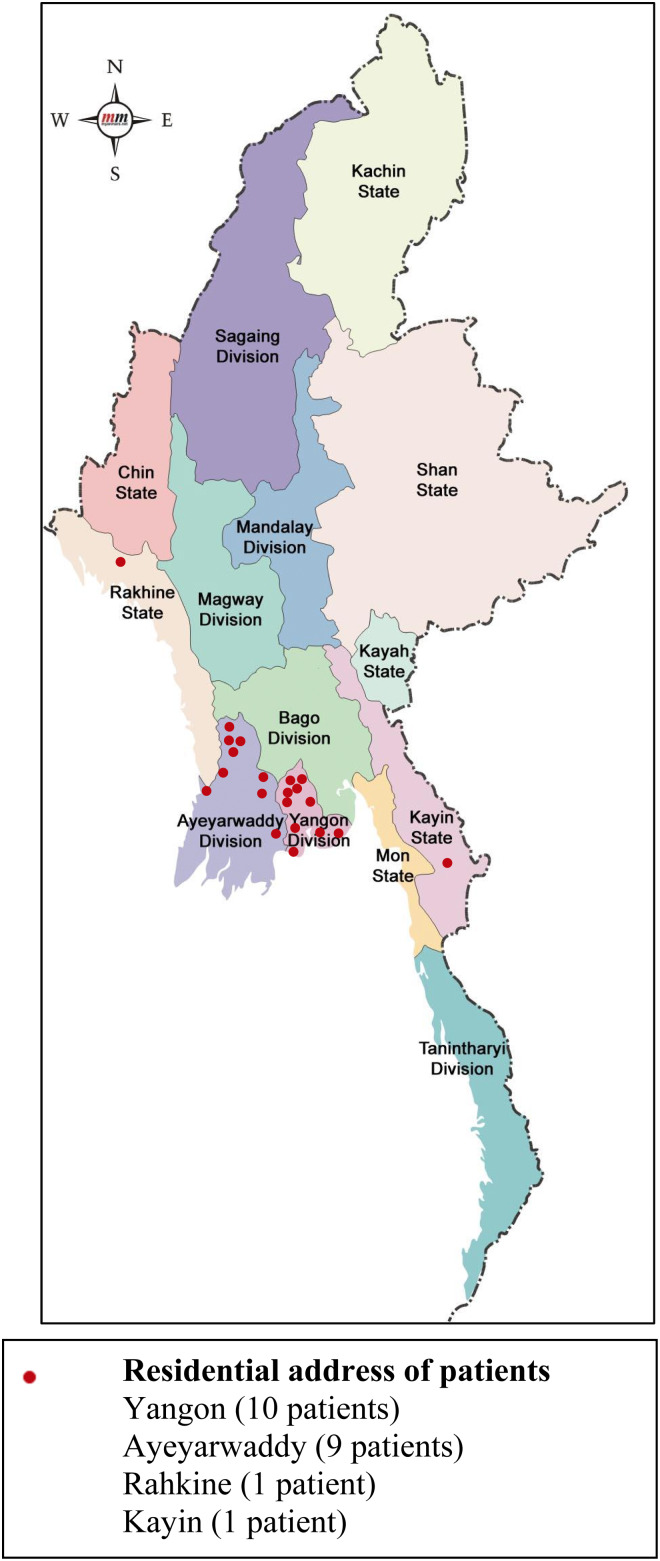
Map showing townships of residence of 21 melioidosis patients. Source :https://images.app.goo.gl/MRMRp1xtJcXbghx46.

Twenty-one of 364 oxidase-positive Gram-negative rod isolates (5.7%) from 21 individual patients were identified as *B. pseudomallei*. Basic clinical and demographic data for these patients are shown in Table 1. Twelve were male, with a median age of 55 years (range 18–79), and nine were female, with a median age of 51 years (range 21–61). Nineteen patients were diagnosed at YGH, one each from IGH and NOGH and none at TGH. Ten patients were from Yangon Region, nine were from Ayeyarwaddy Region, and one patient each was from Kayin and Rahkine States (Fig. 1).

**Table 1.** Clinical and demographic data for 21 patients with culture-confirmed melioidosis

Sr no	Admission diagnosis	Age	Gender	Positive sample	Residence − state or region (township)	Underlying disease	Outcome	Hospital
1	Tetanus	51	Male	Blood	Ayeyarwaddy (Danuphyu)	Nil known	Discharged	YGH
2	Cerebrovascular accident, hypertension, diabetes mellitus	70	Male	Suction tip	Yangon	Diabetes mellitus	Discharged	YGH
3	Septicaemia	70	Male	Blood	Ayeyarwaddy	Diabetes mellitus	Discharged	YGH
4	Suspected lung carcinoma with pneumonia	57	Female	Sputum	Yangon (Thanlyin)	Nil known	Discharged	YGH
5	Liver abscess	39	Male	Blood	Yangon (Thaikkyi)	Nil known	Died	YGH
6	Septicaemia	53	Female	Blood	Yangon	Diabetes mellitus	Discharged	YGH
7	Pneumonia	50	Female	Sputum	Yangon	Nil known	Self discharged	IGH
8	Septicaemia	49	Male	Blood	Rahkine	Renal insuffciency	Died	YGH
9	Septicaemia	66	Male	Blood	Yangon	Nil known	Discharged	YGH
10	Septicaemia	42	Male	Blood	Ayeyarwaddy	Nil known	Discharged	NOGH
11	Septicaemia	56	Female	Blood	Ayeyarwaddy	Diabetes mellitus	Discharged	YGH
12	Aspiration pneumonia	79	Male	Sputum	Kayin	Past tuberculosis of lung	Discharged	YGH
13	Septicaemia	18	Male	Blood	Ayeyarwaddy	Nil known	Discharged	YGH
14	Septicaemia	42	Female	Blood	Ayeyarwaddy	Nil known	Discharged	YGH
15	Septicaemia	62	Female	Blood	Ayeyarwaddy	Diabetes mellitus	Discharged	YGH
16	Septicaemia	21	Female	Blood	Ayeyarwaddy	Nil known	Discharged	YGH
17	Urinary tract infection	48	Female	Urine	Ayeyarwaddy	Nil known	Discharged	YGH
18	Wound infection	58	Male	Wound swab	Yangon	Nil known	Discharged	YGH
19	Wound infection	74	Male	Wound swab	Yangon	Nil known	Discharged	YGH
20	Wound infection	51	Female	Wound swab	Yangon	Nil known	Discharged	YGH
21	Urinary tract infection	35	Male	Urine	Yangon	Nil known	Discharged	YGH

Abbreviations:

YGH = Yangon General Hospital, IGH = Insein General Hospital, NOGH = North Okklapa General Hospital.

Of the 21 patients, a history of soil contact was given by seven, five were reported as having diabetes mellitus and one was reported as having renal insufficiency. The patients presented with septicaemia (12 patients), pneumonia (three patients), urinary tract infection (two patients) and wound infection (four patients). Among the septicaemic patients, two died and one was taken home from hospital in a severe condition and was not expected to survive, but the remaining 18 patients all recovered and were subsequently discharged (Table 1).

All nine isolates tested by *B. pseudomallei* specific PCR were confirmed as *B. pseudomallei.* BOX typing of seven of nine clinical strains demonstrated diversity, with multiple BOX patterns observed (Figure S1). Furthermore, the BOX patterns of the clinical strains did not match the BOX patterns of any of seven Myanmar environmental strains tested simultaneously, demonstrating no link between the environmental and patient strains, which aligns with the epidemiological data (Figure S1).

Following repeat testing where appropriate, testing of the 21 *B. pseudomallei* isolates by disc diffusion in Yangon found one to have intermediate susceptibility to chloramphenicol and one intermediate susceptibility to ceftazidime. Testing of the first seven isolates against selected antibiotics by gradient diffusion in Darwin did not confirm any of the resistances that had been suspected following initial tests in Yangon (Table S2).

## Discussion

Melioidosis is a potentially fatal disease caused by *B. pseudomallei*, which requires specific and prolonged antibiotic treatment to prevent death and relapse. The infection has been recognised increasingly throughout the tropics over the past 40 years, although underdiagnosis remains a significant problem due to a lack of clinical awareness, a lack of familiarity with the organism amongst laboratory staff and the under-development of diagnostic services serving the poor rural population most likely to be affected [3]. The sensitivity of culture for *B. pseudomallei* from clinical specimens can be improved with the use of selective media [22]. However, even with positive cultures, the organism is frequently misidentified or disregarded as a contaminant [23]. Although not officially recognised as a ‘Neglected Tropical Disease’ (NTD), it has been estimated that melioidosis may cause a greater burden of disability-adjusted life-years (DALYs) than many recognised NTDs such as dengue, schistosomiasis and leishmaniasis [24]. Even in countries where its importance has been highlighted by researchers, such as Thailand, official statistics have failed to capture the true disease burden, especially in terms of mortality [25].

Myanmar has a special place in the history of melioidosis, as it is where the disease was first recognised by Alfred Whitmore and CS Krishnaswami working in Rangoon in 1911 [1, 2]. However, apart from a single case report in 1948 [26], the disease ‘disappeared’ from the Myanmar medical literature until the beginning of the 21st century [8] and since then only a handful of cases has been reported [27]. We therefore undertook this study in four tertiary hospitals in Yangon, including that in which melioidosis had first been identified, in order better to understand the true incidence of meliodosis in modern day Yangon. We attempted to improve the awareness of the disease amongst clinicians and laboratory staff in the respective hospitals prior to the study in order to encourage submission of relevant diagnostic specimens to their laboratories, and to overcome the difficulties in the identification of *B. pseudomallei* by collecting all oxidase-positive Gram-negative bacilli for identification in a central laboratory.

Despite this, we only identified 21 *B. pseudomallei* out of 364 isolates tested (5.7%) over a 2-year period. It is notable that all but two of these were detected at YGH, the largest hospital with the most complex casemix and one of only two of the hospitals in the study with automated systems for blood cultures and bacterial identification. *B. pseudomallei* had, however, previously been reported to account for only one of 90 episodes of bacteraemia in YGH in a one year period between 2015 and 2016 [28]. In another study reported in 2013, only three *B. pseudomallei* isolates were obtained from 85 pus and wound swab samples (3.5%) in YGH [12]. Overall, the incidence of confirmed melioidosis in Yangon was not high compared with parts of neighbouring endemic countries such as Thailand, Cambodia and Laos [29–31]. It thus appears that melioidosis may not now be as common in Yangon as it was in the time of Krishnaswamy, who in 1917 reported seeing more than 200 cases over 6 years in the mortuary of YGH, accounting for one in every 20 autopsies he performed [7]. There are several possible explanations for this, most notably the fact that all four hospitals were within the city of Yangon, which is considerably more urbanised now than it was in the early part of the 20th century, when there were still paddy fields in what is now the centre of a city with over 5 million inhabitants.

In terms of the clinical and epidemiological features of the 21 patients in our series, there appears to be nothing particularly unusual about melioidosis in modern day Myanmar to distinguish it from other parts of south east Asia [29–31], apart from the relatively low mortality [3 of 21 (14%) patients], which suggests that some more severe cases may have been being missed. Unfortunately, we do not have any follow-up data and so cannot be certain whether any patients died or relapsed following hospital discharge. It is worthy of note that only 33.3% of our patients reported an occupation in agriculture, although most of the patients resided in Ayeyarwaddy and outskirts of Yangon (nine and ten cases, respectively), where a high proportion of the population are involved in farming. Even if patients do not report their occupation as ‘farmer’, many families have some land and engage in agricultural activities such as rice farming some of the time. Diabetes was a common predisposing factor, reported in five of our patients, although this may have been under-recognised or under-reported. The prevalence of diabetes mellitus, the major risk factor for melioidosis, is rapidly increasing in Myanmar [32]. There were also no patients who were injecting drug users in our series, whereas the majority (31/38) of the original melioidosis cases described by Whitmore and Krishnaswami, and 95% of those later reported by Krishnaswamy, showed evidence of being morphia injectors leading to the nickname ‘morphia injector's septicaemia’ [2, 7]. Although occasional reports of melioidosis in injecting drug users have appeared subsequently [33], this has not featured as a major risk factor for the disease since the early reports from Myanmar. The reason for this particular historic association remains as yet unexplained, although it is likely that it is accounted for by specific behavioural or environmental factors amongst morphine injectors in early 20th century Yangon and possibly the more intensive microbiological investigation of patients who underwent *post mortem* examination as ‘police cases’. Interestingly, there is speculation that some of the more recent unexplained autochthonous cases of melioidosis seen in the USA may be the result of potential contamination of medical commodities, including intravenous products, imported from Southeast Asia [34].

As far as the microbiology of melioidosis in Myanmar is concerned, again the isolates of *B. pseudomallei* appeared relatively typical for the species. On the basis of disc diffusion testing carried out in Yangon, it initially appeared that there was more resistance to antibiotics usually used to treat melioidosis, particularly beta-lactams, than would generally be expected in *B. pseudomallei* (data not shown). The prolonged antibiotic regimens used in melioidosis can lead to *B. pseudomallei* acquiring resistance, although the frequency of co-trimoxazole resistance in *B. pseudomallei* has previously been over-estimated by disc diffusion testing [35]. Ceftazidime resistance has been widely reported in patients following treatment but is not common [36], and meropenem resistance is exceedingly rare. On re-testing in Yangon, however, the majority of these initial results could not be confirmed and they were assumed to be related to a loss of disc potency on the initial testing. In addition, none of these initial results could be confirmed when seven isolates were re-tested in Darwin by the gradient strip method. Molecular characterisation of nine of the isolates from our study is reported in detail elsewhere [21]. However, in summary, phylogenomic analysis of *B. pseudomallei* genomes from Myanmar in the context of a global set of *B. pseudomallei* genomes demonstrated that Myanmar *B. pseudomallei* reside within the Asian clade and cluster with genomes from countries bordered by the Mekong River. Noteworthy is that Myanmar *B. pseudomallei* isolates are very diverse with numerous sequence types (STs) detected, and our BOX-PCR results align with the noted high diversity [20, 21] (Figure S1).

The main limitation of our study is that it is likely that our enhanced laboratory-based surveillance will still have missed patients with melioidosis. Patients with fevers, pneumonia and abscesses are not always investigated intensively for melioidosis in Myanmar, and selective media such as Ashdown's agar, which are known to increase the yield of culture for *B. pseudomallei* from sites with a normal flora, were not used for primary isolation in this study because of resource constraints [22]. In addition, by restricting our surveillance to hospital laboratories and not including private laboratories, even culture-positive cases admitted to the four hospitals during the study may have been missed. This is because microbiology services in Myanmar are fragmented and hospital laboratories are currently only open during standard hours of work (8:00 AM to 5:00 PM). Samples collected outside these hours are usually sent to private laboratories and clinicians may also opt to send samples to private laboratories at other times. We are aware of at least two patients with culture-positive melioidosis at IGH who were diagnosed by private laboratories during our study period but were missed by our surveillance for this reason, and it is likely that similar instances occurred at the other hospitals.

Overall, it is clear that melioidosis is still endemic in Yangon and neighbouring provinces, although considerable further work will be needed to establish the true disease burden. Furthermore, it is likely that there will be areas of much higher incidence outside Yangon. For example, melioidosis is relatively uncommon in hospitals in Bangkok compared with hospitals in northeast Thailand where it is a major cause of community-acquired sepsis [30]: similar hotspots may well exist in rural Myanmar. Further studies are therefore necessary to determine the true distribution of *B. pseudomallei* and incidence of melioidosis at selected locations in Myanmar. Since rice farming is a frequent activity in suburban and rural areas in Myanmar, it is on such populations that efforts should initially be focused. Further work is also needed at a national level to raise awareness of the disease amongst clinicians and laboratory staff to enable patients to be effectively diagnosed and appropriately treated.

## Supplementary material

For supplementary material accompanying this paper visit http://dx.doi.org/10.1017/S095026882100128X. click here to view supplementary material

## Data

The data that support the findings of this study are available from Dr Mo Mo Win on request.
