# Supplementary material for: Enhanced melioidosis surveillance in patients attending four tertiary hospitals in Yangon, Myanmar
Source: Epidemiol Infect. 2021 Jun 22;149:e154. doi: 10.1017/S095026882100128X (PMC8276317; doi:10.1017/S095026882100128X)

**Supplementary Material**

*Epidemiology and Infection*

**Enhanced melioidosis surveillance in patients attending four tertiary hospitals in Yangon, Myanmar**

Mo Mo Win *et al*.

**Supplementary Material 1 Data Collection Proforma**

Detection of *Burkholderia* *pseudomallei* in patients attending Yangon, Thingungyun, Insein and North Okklapa General Hospital

**Identification**

Name……………………………………………………………….. Age …... Sex…..

Occupation………………………………………………………………..………………………..

Address……………………………………………………………..………………………………

Hospital No..…………………………….. Unit & Ward …...…………………………………..

Admission; Date ….…. / ..…… / …..… Time ………..

Provisional diagnosis………………………………….………………………….………………..

Date confirmed as melioidosis…………………………………………………………………….

**Main clinical features (tick all that apply)**

Septic shock ☐

Pneumonia ☐

Liver abscess ☐

Splenic abscess ☐

Kidney abscess ☐

Prostate abscess ☐

Lymphadenitis ☐ If yes, site …………………………..……………..

Other abscess ☐ If yes, site ……………………….…..…………….

Skin/soft tissue infection ☐ If yes, site ………………………………………….

Osteomyelitis ☐ If yes, site …………………………..……………..

Septic arthritis ☐ If yes, site …......…………………..………………

Other (please give details) …………………………………………………………...…………….

………………………………………………………………………………………………………**Underlying diseases (tick all that apply)**

Thalassemia ☐

Diabetes ☐

Renal failure ☐

Renal calculi ☐

Liver disease (Chronic hepatitis, cirrhosis of liver) ☐

Malignancy ☐ If yes, specify type……………………………………

Alcohol abuse ☐ If yes, units/week............. duration……………............

Other narcotics abuse ☐ If yes, agent ............. duration........................................

Steroid therapy ☐ If yes, dose ……….. duration ………………………….

Other (please give details) ……………………………………………………………….

**Antibiotic treatment**

1. *Prior to diagnosis*

Agent:…………………………………… Dose:……………………… Duration:……days

Agent:…………………………………… Dose:……………………… Duration:……days

1. *After diagnosis*

Intensive phase

Agent:…………………………………… Dose:……………………… Duration:……days

Agent:…………………………………… Dose:……………………… Duration:……days

1. Eradication phase

Agent:…………………………………… Dose:……………………… Duration:……days

Agent:…………………………………… Dose:……………………… Duration:……days

**Risk Factors**

Contact with animals ☐ If yes, please give details:………………………………………….…

Contact with soil ☐ If yes, please give details:……………………………………………

Contact with water ☐ If yes, please give details:…………………………………………..…..

**Outcome**

Discharged well ☐ Discharged moribund ☐ Died in hospital ☐

**Any other relevant information:**

……………………………………………………………………………………………………

…………………………………………………………………………………………………….

…………………………………………………………………………………………………….

#### Supplementary Table S1.

#### MICRO framework - A checklist of items that should be addressed in reports of studies involving human clinical microbiology data

#### [Core “must include” items are indicated by an asterisk]

| Section | Item No | Recommendation | Completed  Yes / No / NA | Page No |
| --- | --- | --- | --- | --- |
| Methods |  |  |  |  |
| *Study design* | 1* | **Specimen types**: Describe the types of specimen included, i.e. clinical (e.g. blood cultures) or non-diagnostic surveillance (e.g. admission and other screening swabs to diagnose carriage). If specimens were obtained for diagnostic reasons, clinical syndromes should be described where possible, and specimens / isolates stratified by clinical syndrome. | Yes | 2 |
|  | 2* | **Sampling period:** State the collection timeframe for specimens yielding isolates for which data is reported, e.g. from MM/YY to MM/YY to be able to identify variability between seasons. | Yes | 4 |
|  | 3* | **Sampling strategy:** Describe the strategy for specimen collection, e.g. asymptomatic screening, sampling of all febrile patients, sampling at clinician discretion, sampling of specific patient groups, convenience sampling (e.g. use of isolates from an existing sample repository). Specify whether sampling followed routine clinical practice or was protocol driven. Classify specimens as from community-acquired (CAI) or hospital-acquired (HAI) infections. The definition of HAI used (e.g. HAI defined by specimen collection >48h after hospital admission) should be provided and should use ideally an international standard (e.g. US-Centers for Disease Control (1, 2)). | Yes (physician discretion after awareness raising activities) | 5 |
|  | 4 | **Target organisms:** Explicitly state which organisms / organism groups were included in the report. Nomenclature should follow international standards (i.e. using approved genus / species names as summarised in the International Journal of Systematic and Evolutionary Microbiology). Lists of approved bacterial names can be downloaded from [Prokaryotic Nomenclature Up-to-Date](https://www.dsmz.de/bacterial-diversity/prokaryotic-nomenclature-up-to-date.html) and the [List of Prokaryotic Names with Standing in Nomenclature](http://www.bacterio.net/). Organisms considered contaminants should be listed, if appropriate (e.g. coagulase negative staphylococci or *Corynebacterium* spp. (3, 4)). | Yes (*Burkholderia pseudomallei*) | 5 |
| *Setting* | 5* | **Geographical setting:** Describe the geographical distribution of specimens / patients from which isolates were obtained; at least to a country level, but preferably to a sub-national level or a geoposition. | Yes (State or Region) | 7 |
|  | 6* | **Clinical setting:** Describe the type and level of the healthcare facilities (e.g. primary, secondary, tertiary) from which specimens were obtained. If stating a microbiology laboratory, the centres served by the laboratory should be specified. | Yes | 4 |
| *Laboratory work* | 7 | **Specimen processing:** If applicable, describe specimen collection and handling, processing and sub-culture methods for all types of specimen included. For example, if reporting AST results for blood culture and cerebrospinal fluid culture isolates, the processing of these specimens by the laboratory should be briefly explained, including how specimens are sub-cultured, the media used, incubation conditions and duration. A summary of specimen processing steps (e.g. pre-processing steps, nucleic acid extraction method (if applicable), amplification platform, contamination avoidance strategy) should be provided for molecular-only workflows (e.g. to detect *Mycobacterium tuberculosis* and rifampicin resistance using the Cepheid Xpert MTB / RIF system). | Yes (and SOP referenced) | 6 |
|  | 8* | **Target organism identification:** Details of identification methodology should be reported briefly. Where identification databases were used (e.g. bioMerieux API / bioMerieux VITEK-MS / Bruker Biotyper), the version should be specified. In general, all pathogens should be identified to species level. In the case of *Salmonella* species, organisms should be identified to at least the *S.* Typhi, *S.* Paratyphi, or non-typhoidal salmonella (NTS) level. Strain subtyping methods should be reported according to STROME-ID (5). | Yes (API) | 6 |
|  | 9* | **Antimicrobial susceptibility testing:** Describe the antimicrobial susceptibility testing methods used, internal quality control processes, and their interpretation, with reference to a recognised international standard – e.g. CLSI, EUCAST. Where an international standard was followed, the specific edition(s) of guidelines used should be referenced. Deviations from standard methodology should be described, along with evidence of validation. Handling of any changes to interpretative criteria during the sampling period should be documented. State whether the raw AST data (zone diameters and / or minimum inhibitory concentrations) were re-categorised with updated breakpoints or left as-is. | Yes (CLSI 2017) | 6 |
|  | 10 | **Additional tests performed to identify resistance mechanisms:** Describe the testing methods used for adjunctive / confirmatory antimicrobial susceptibility tests, such as enzymatic / molecular assays (e.g. Xpert MTB / RIF, mecA PCR) and inducible resistance assays, with reference to a recognised international standard, where available. Where an international standard was followed, the specific edition of guidelines used should be referenced. Deviations from standard methodology should be described, along with evidence of validation. | NA |  |
|  | 11* | **Antimicrobial resistance definitions:** Define resistance for each antimicrobial class (i.e. are isolates in the “intermediate” category included within “susceptible” or “resistant” or analysed as a distinct category). If using the term, define MDR (e.g. ≥1 agent in ≥3 classes tested). For each organism type, an MDR test panel must be defined, consisting of the minimum panel of individual antimicrobial agents / classes against which an isolate must be tested for that isolate to be considered tested for MDR status. Antimicrobials to which an organism is intrinsically resistant cannot be part of the test panel or contribute to MDR status (6, 7). | Yes (intermediate using CLSI guidance) | 8 and Table S1 |
| *Quality assurance* | 12* | **External quality assurance:** State whether the microbiology laboratory participates in an external quality control programme and, if so, provide scheme details. Examples include the [UK National External Quality Assurance Scheme](http://www.ukneqasmicro.org.uk) and the [American College of Pathologists External Quality Assurance / Proficiency Testing Program](https://www.cap.org/laboratory-improvement/international-laboratories/external-quality-assurance-proficiency-testing-for-international-laboratories). | No |  |
|  | 13 | **Accreditation:** State whether the laboratory is accredited through a national or international body (e.g. the International Standards Organisation, ISO) and specify which assays are covered in the accreditation. | No |  |
| *Bias* | 14* | **Duplicate and sequential isolates:** The strategy for accounting for duplicate and sequential isolates from the same patient should be clearly detailed. Duplicate isolates are multiple isolates of the same phenotypic organism (i.e. same species and same resistance profile) from the same patient on the same date cultured either from the same clinical specimen, or from two separate clinical specimens, such as blood and CSF. Sequential isolates are isolates of the same phenotypic organism from the same patient at different dates, such as blood cultures taken on different dates. Various strategies for the handling of duplicate and sequential isolates exist (8), and the strategy used should be transparent as it will bias pooled resistance results. For example, inclusion of all isolates (the ‘all isolate strategy’), has been shown to shift pooled resistance proportions toward greater resistance, whilst inclusion of only the first isolate per patient (the ‘first isolate strategy’) or only the first isolate per infection episode (the ‘episode-based strategy’) will shift pooled results towards susceptibility. | NA |  |
| Results |  |  |  |  |
|  | 15* | **Population:** Describe the demographics of the population from which clinical specimens and subsequent isolates have been obtained, disaggregating age and gender data. | Yes | Table 1 |
|  | 16* | **Denominators:** Patient and isolate denominators should be used appropriately to ensure clarity regarding the numbers included in each analysis. Of particular importance is the reporting of resistance where first- and second-line AST panels were used (i.e. not all isolates of a particular species were tested against all agents). For drugs where only a subset of isolates were tested, reporting of a percentage without the numbers of isolates tested / resistant may be highly misleading. | Yes | Table S1 |
|  | 17 | **Site / place of acquisition**: AST data from CAI and HAI should be reported and analysed separately. | NA (all CAI) |  |
|  | 18* | **Reporting resistance proportions for single agent and class resistance:** Proportions of resistant isolates should be reported as number of isolates susceptible or resistant to a given antimicrobial agent / class out of actual number of isolates tested for susceptibility to that agent / class. | NA |  |
|  | 19 | **Reporting multidrug resistance proportions:** If defined, the proportion of MDR isolates should be expressed as the number of MDR isolates out of the number of isolates tested (i.e. the number undergoing the MDR test panel specific to that organism). Single agent / class resistance should be always be reported, regardless of MDR reporting. | NA |  |
| Discussion |  |  |  |  |
| *Limitations* | 20 | Discuss any reasons why bias may have been introduced into the reported data, due to patient / specimen selection, isolation of organisms, or otherwise. Consider factors which may have either introduced bias into the types of organisms isolated or the antimicrobial susceptibility profiles, e.g. receipt of antimicrobials prior to specimen collection will reduce the yield of certain species and also select for more resistant organisms. | Yes | 12 |

**Note**

It is recommended that this checklist is used in conjunction with the original article (9), available on the Web site of BMC Medicine at <https://bmcmedicine.biomedcentral.com/articles/10.1186/s12916-019-1301-1> (DOI: <https://doi.org/10.1186/s12916-019-1301-1>). The checklist is reproduced from this article under the terms of the Creative Commons Attribution 4.0 International License (CC BY 4.0; <http://creativecommons.org/licenses/by/4.0/>).

**References**

1. CDC/NHSN. Identifying Healthcare-associated Infections (HAI) for NHSN Surveillance 2018. Available from: <https://www.cdc.gov/nhsn/pdfs/pscmanual/2psc_identifyinghais_nhsncurrent.pdf>.

2. CDC/NHSN. Surveillance Definitions for Specific Types of Infections 2018. Available from: <https://www.cdc.gov/nhsn/pdfs/pscmanual/17pscnosinfdef_current.pdf>.

3. Hossain B, Islam MS, Rahman A, Marzan M, Rafiqullah I, Connor NE, et al. Understanding Bacterial Isolates in Blood Culture and Approaches Used to Define Bacteria as Contaminants: A Literature Review. Pediatr Infect Dis J. 2016;35(5 Suppl 1):S45-51.

4. Hossain B, Weber MW, Hamer DH, Hibberd PL, Ahmed AS, Marzan M, et al. Classification of Blood Culture Isolates Into Contaminants and Pathogens on the Basis of Clinical and Laboratory Data. Pediatr Infect Dis J. 2016;35(5 Suppl 1):S52-4.

5. Field N, Cohen T, Struelens MJ, Palm D, Cookson B, Glynn JR, et al. Strengthening the Reporting of Molecular Epidemiology for Infectious Diseases (STROME-ID): an extension of the STROBE statement. Lancet Infect Dis. 2014;14(4):341-52.

6. Magiorakos AP, Srinivasan A, Carey RB, Carmeli Y, Falagas ME, Giske CG, et al. Multidrug-resistant, extensively drug-resistant and pandrug-resistant bacteria: an international expert proposal for interim standard definitions for acquired resistance. Clin Microbiol Infect. 2012;18(3):268-81.

7. German GJ, Gilmour M, Tipples G, Adam HJ, Almohri H, Bullard J, et al. Canadian recommendations for laboratory interpretation of multiple or extensive drug resistance in clinical isolates of Enterobacteriaceae, *Acinetobacter* species and *Pseudomonas aeruginosa*. Can Commun Dis Rep. 2018;44(1):29-34.

8. Hindler JF, Stelling J. Analysis and presentation of cumulative antibiograms: a new consensus guideline from the Clinical and Laboratory Standards Institute. Clin Infect Dis. 2007;44(6):867-73.

9. Turner P, Fox-Lewis A, Shrestha P, Dance DAB, Wangrangsimakul T, Cusack TP, et al. Microbiology Investigation Criteria for Reporting Objectively (MICRO): a framework for the reporting and interpretation of clinical microbiology data. BMC Med. 2019;17(1):70.

**Table S2: Antimicrobial susceptibility results for 21 Myanmar clinical *B. pseudomallei* isolates.**

| ***B. pseudomallei* strain** | **DMR disc diffusion zone diameter/category**  **(Results in brackets are Darwin gradient diffusion MIC results, where available)** | | | | | |
| --- | --- | --- | --- | --- | --- | --- |
|  | **DOX** | **SXT** | **CHL** | **CAZ** | **AMC** | **MEM** |
| Y 3 | 28/S | 20/S | 18/I | 18/S  (1/S) | 19/S | 19/S |
| Y 21 | 26/S | 25/S | 28/S | 21/S  (1.5/S) | 20/S (2) | 34/S |
| Y 22 | 28/S | 25/S | 28/S | 28/S | 28/S | 22/S  (0.75/S) |
| Y 41 | 29/S | 25/S | 25/S | 29/S | 31/S | 23/S  (0.75/S) |
| Y 42 | 23/S | 18/S | 21/S | 25/S | 30/S | 19/S |
| M 72 | 33/S | 32/S | 31/S | 21/S  (1/S) | 33/S | 33/S |
| M 92 | 22/S | 25/S | 30/S | 28/S | 25/S | 18/S  (1/S) |
| Y 33 | 25/S | 28/S | 30/S | 30/S | 28/S | 30/S |
| Y 43 | 25/S | 33/S | 27/S | 27/S | 28/S | 23/S  (0.75/S) |
| Y 44 | 25/S | 27/S | 29/S | 25/S | 27/S | 30/S |
| Y 46 | 27/S | 16/S | 23/S | 25/S | 32/S | 23/S |
| Y 47 | 21/S | 18/S | 27/S | 32/S | 30/S | 27/S |
| Y 48 | 28/S | 31/S | 26/S | 35/S | 32/S | 21/S |
| Y 49 | 30/S | 28/S | 27/S | 25/S | 28/S | 19/S |
| Y 50 | 25/S | 32/S | 25/S | 23/S | 32/S | 21/S |
| Y 51 | 28/S | 31/S | 28/S | 32/S | 33/S | 27/S |
| Y 52 | 35/S | 28/S | 32/S | 16/I | 36/S | 23/S |
| Y 104 | 22/S | 34/S | 20/S | 30/S | 34/S | 30/S |
| Y 110 | 32/S | 27/S | 28/S | 23/S | 32/S | 28/S |
| Y 135 | 18/S | 25/S | 22/S | 27/S | 35/S | 32/S |
| Y 151 | 24/S | 32/S | 28/S | 18/S | 32/S | 23/S |

DMR, Department for Medical Research, Yangon; MIC, minimum inhibitory concentration; S, susceptible; I, intermediate; R, resistant; DOX, doxycycline; SXT, trimethoprim-sulfamethoxazole; CHL chloramphenicol; CAZ, ceftazidime; AMC, amoxicillin-clavulanic acid; MEM, meropenem

**Figure S1: A BOX-PCR of 9 clinical and 7 soil *B. pseudomallei* isolates from Myanmar, with molecular weight markers on the outside lanes.** **DNA bands for isolates according to their size are represented by white and red lines. The seven soil isolates are not epidemiologically linked to the seven clinical isolates, but were included here for comparison and to investigate whether any shared the same BOX typing patterns.**


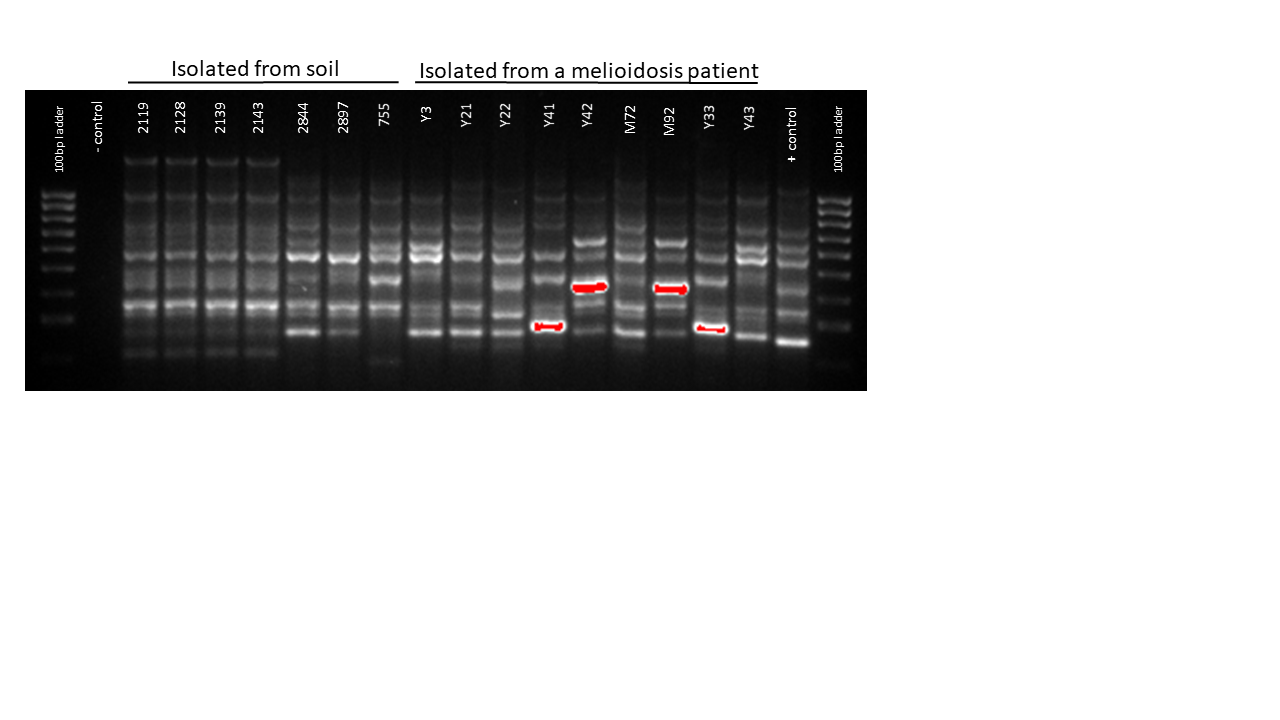

Supplement: Supplementary file 1 [file S095026882100128Xsup001.docx]
